# Systematic Analysis of Sex-Linked Molecular Alterations and Therapies in Cancer

**DOI:** 10.1038/srep19119

**Published:** 2016-01-12

**Authors:** Jonathan Ma, Sadhika Malladi, Andrew H Beck

**Affiliations:** 1 The Harker School, CA 95128; 2 Department of Pathology, Beth Israel Deaconess Medical Center and Harvard Medical School, MA.

## Abstract

Though patient sex influences response to cancer treatments, little is known of the molecular causes, and cancer therapies are generally given irrespective of patient sex. We assessed transcriptomic differences in tumors from men and women spanning 17 cancer types, and we assessed differential expression between tumor and normal samples stratified by sex across 7 cancers. We used the LincsCloud platform to perform Connectivity Map analyses to link transcriptomic signatures identified in male and female tumors with chemical and genetic perturbagens, and we performed permutation testing to identify perturbagens that showed significantly differential connectivity with male and female tumors. Our analyses predicted that females are sensitive and males are resistant to tamoxifen treatment of lung adenocarcinoma, a finding which is consistent with known male-female differences in lung cancer. We made several novel predictions, including that CDK1 and PTPN1 knockdown would be more effective in males with hepatocellular carcinoma, and SMAD3 and HSPA4 knockdown would be more effective in females with head and neck squamous cell carcinoma. Our results provide a new resource for researchers studying male-female biological and treatment response differences in human cancer. The complete results of our analyses are provided at the website accompanying this manuscript (http://becklab.github.io/SexLinked).

Sex is widely recognized as an important factor in the progression of cancer. However, few studies of cancer effectively incorporate sex differences into their study design. For example, only 20% of guinea pigs used in animal testing are female^1^; furthermore, the Food and Drug Administration (FDA) excluded women from phase I and II human clinical trials starting in 1977, only rescinding this policy in 1993^1^. Even today, women comprise only 37% of trial participants in nine major medical journals^2^. Thus, females are historically underrepresented in pre-clinical and clinical studies of cancer, leading to a dearth of knowledge regarding sex disparities in cancer progression and treatment.

Furthermore, although the efficacies of several existing anticancer drugs have been shown to differ between the sexes^3^,^4^,^5^, identifying drugs with efficacy that varies across the sexes using conventional guess-and-check approaches is time consuming, expensive, and not scalable. Thus, there would be tremendous value in the development of a systematic data-driven approach for identifying significant molecular differences in tumors in men and women, and then to use this knowledge to inform the rational prioritization of cancer drugs predicted to show differential efficacy based on sex.

The goal of our study is to identify transcriptomic differences between tumors in males and females, and to use this knowledge to inform drug sensitivity predictions in male and female tumors. In order to predict sex-disparate drug sensitivity, we developed a computational framework consisting of three steps: identification of genes that show differential expression in cancer with respect to patient sex; identification of genes and pathways differentially expressed between tumor and normal samples stratified by sex; and use of sex-specific gene expression signatures to prioritize chemical and genetic perturbagens predicted to have differential efficacy in males vs. females (Fig. 1).

## Results

### Transcriptome wide univariate analysis of differential expression by sex across 17 cancers

Our transcriptomic analyses used gene expression (RNAseqV2) data downloaded from The Cancer Genome Atlas (TCGA)^6^ for a total of 17 cancers. A table containing those cancers and the abbreviations used to refer to them is available as Table S1. We computed the non-parametric Wilcoxon Rank-Sum Test with respect to sex within each cancer, and we adjusted the resulting *p*-values for multiple hypothesis testing using the Benjamini-Hochberg method, yielding Benjamini-Hochberg (BH) values. Within each cancer, we identified genes which exhibited significantly (BH < 0.05) sex-disparate expression, ranging from 28 genes in kidney chromophobe (KICH) to 1,669 genes in kidney renal clear cell carcinoma (KIRC) (Fig. 2). Moreover, for the 7 cancers which had at least 10 normal patients, we identified a total of 4111 genes with significantly sex-differentiated expression in at least one tumor type but not in corresponding normal samples of the same tissue type.

To assess the extent to which these differences in differential gene expression counts were confounded by sample size, we normalized the differential expression counts by sample size (Fig. 2B). This normalization revealed the highest ratio of number of sex-associated differentially expressed genes per patient for KIRP, LIHC, KIRC, and LUAD. Although THCA was not among the cancers with the most sex-associated differentially expressed genes, THCA did contain a relatively small subset of transcripts with extremely strong levels of sex-associated differential expression (BH < 1E-70, Fig. 2A).

### Analysis of transcripts from the sex-chromosomes in male and female tumors

The proportion of genes differentially expressed with respect to sex originating from the sex chromosomes ranged from 7.07% in KIRC to 76.47% in SKCM (Fig. 2B), which is significantly larger than the total proportion of measured genes originating from the sex chromosomes (4.48%) (All *p*-value for difference in proportions <2.2e-16). Thus, these results show that genes differentially expressed with respect to sex in cancer are enriched for genes on the sex-chromosomes; however, a large number of differentially expressed transcripts are also found on autosomes.

To further assess the combinations of genes most associated with sex across the 17 cancers, we performed LASSO regression in each cancer to build multivariate models to classify male vs. female tumors^7^. We assessed the accuracy of these models using 10-fold cross validation, in which we partitioned our sample into 10 folds. We then performed 10 validations: in each validation we trained a model on samples from 9 of the folds and tested the model on the samples from the held out fold. Thus, the 10-fold cross-validated predictive accuracy represents the performance of our models on data unseen during training. We obtained extremely high 10-fold cross-validated prediction accuracy (cross-validated AUC >0.99) for predicting sex based on gene expression data for all 17 cancers and the 1 meta-analysis across all cancers. This high accuracy was obtained with very few genes (ranging from 2–6 active transcripts) in each model, with all active transcripts belonging to the sex chromosomes. There was a very high-degree of commonality across all the models, with three Y-chromosome genes selected across all 17 cancers (ZFY, EIF1AY, and DDX3Y), RPS4Y1 (also on the Y-chromosome) selected across 15 of 17 cancers, and XIST (on the X-chromosome) selected across 14 of 17 cancers. These results show a large degree of consistency in the most sex-linked transcripts across a diverse set of 17 cancers, with the most sex-linked patterns of expression concentrated on several genes within the Y chromosome and XIST on the X chromosome.

To assess whether this finding was specific to cancer or a general phenomenon shared in tumor and normal samples, we repeated the LASSO regression analysis to classify sex in normal samples from 7 tissue types for which we had at least 10 male and female normal samples, and we again assessed performance using cross-validation. Similar to the cancer samples, in normal samples we obtain very high predictive accuracy (AUC > 0.99) in cross-validation with a small set of genes selected from the sex chromosomes. As in tumors, the genes ZFY, RPS4Y1, DDX3Y were selected in all of the tissue types for the male vs. tumor analyses in normal samples, and RPS4Y1 was selected in the majority of tissue types. XIST was a notable difference between the tumor and normal analyses. XIST was selected in the male vs. female classification model for 14 of 17 cancer types, but was not selected in any of the male vs. female analyses in normal samples, suggesting that differences in XIST expression between male and female tissues emerge during oncogenesis.

### Identification of Pathways Differentially Expressed in Tumor Vs Normal Samples Stratified by Sex

Within each sex for each of the 7 cancers that had at least 10 normal samples in each sex, we identified pathways enriched among genes that showed the largest fold-change difference in expression in tumor vs. normal samples using Ingenuity Pathway Analysis (IPA®, QIAGEN Redwood City, www.qiagen.com/ingenuity) (Table 1).

LUAD, KICH, LIHC, and HNSC showed the highest proportion of significant pathways enriched in the tumor vs. normal analyses from only a single sex. Notably, LIHC, LUAD, and HNSC were also among the cancers with the greatest ratio of number of significantly sex-differentiated genes to sample size (Fig. 2), suggesting that the sex-differentiated gene expression patterns discovered in our transcriptomic analyses may play a role in sex-disparate pathway enrichment in these tumor types. A selection of pathways showing enrichment in exclusively one sex (P < 0.05) and no significant enrichment in the other sex (P > 0.05) are shown in Table 2.

Several pathways associated with LIHC tumors were only significantly associated with one sex but not the other, including Liver X Receptor (LXR) and Retinoid X Receptor (RXR) activation. LXR and RXR, which regulate lipid metabolism^8^, have been identified as therapeutic targets in cancer and metabolic diseases but are only identified as enriched in the tumor vs. normal analysis in males, suggesting that targeting LXR/RXR activation may show the greatest efficacy in LIHC tumors in males. In contrast, the sonic hedgehog (SHH) signaling pathway was significantly enriched in female LIHC tumors but not in males. Thus, our results suggest that inhibition of SHH signaling may be more effective in treating LIHC in females than in males. Intriguingly, EIF2 signaling was significantly enriched in male HNSC tumors and female LUAD tumors, suggesting that this pathway may play a different and sex-disparate role in the two cancers.

### Connectivity Map Analysis

The LincsCloud Connectivity Map (http://www.lincscloud.org) is an online resource to link disease-associated transcriptional signatures with chemical and genetic perturbagen-associated transcriptional signatures. In our analysis, we used LincsCloud to link cancer-associated signatures with chemical and genetic perturbagens in sex-stratified analyses. We made predictions of sex-disparate drug efficacy using this connectivity map analysis in an effort to identify 3 types of perturbagens: Type I, to which males are sensitive and females are resistant; Type II, to which females are sensitive and males are resistant; and Type III, to which both sexes are sensitive. We used permutation analysis to compute sex-differential p-values and corrected for multiple hypotheses using the method of Benjamini and Hochberg. We required permutation-based BH value (BH < 0.05) for perturbagens to be classified as Type I or II and an insignificant BH value (BH > 0.95) and connectivity scores greater than or equal to 90 in both sexes to be considered as Type III.

To create our input signatures, we first selected genes which exhibited significant (BH<0.05) differential expression between tumors and normal in sex-stratified analyses, and from those genes we used tumor-normal expression fold change to rank the genes and selected the top 250 genes in each of four categories: up in male tumors (male resistance signature), up in female tumors (female resistance signature), up in male normals (male sensitivity signature), up in female normals (female sensitivity signature). We queried the LincsCloud connectivity map analysis tool with our input signatures to obtain connectivity scores for perturbagens. A strongly positive mean connectivity score for a perturbagen in one sex indicated that tumor cells of that sex were predicted to be sensitive to the perturbagen; conversely, a strongly negative mean connectivity score indicated that tumor cells of that sex were predicted to be resistant. Thus, Type I perturbagens exhibit strongly positive male and negative female connectivity scores, Type II perturbagens exhibit strongly negative male and positive female scores, and Type III perturbagens exhibit strongly positive scores in both sexes. We employed a permutation-based statistical approach inspired by the PMASE method of Liu *et al.*^9^, in which we created *N* = 1,000 random permutations of the patient gender at the sample level, and performed connectivity map queries using signatures constructed from the permuted data sets. We then computed a null distribution of male-female perturbagen difference scores on the permuted dataset. We used these null distributions to compute raw *p*-values for the observed male-female perturbagen differences, and we adjusted the raw *p*-values for multiple hypotheses using the method of Benjamini and Hochberg.

Separately within each sex, we obtained mean connectivity scores of over 42,000 perturbagens (~22,000 genetic reagents and ~20,000 chemical reagents) within each cancer. In total, our method yielded 493 Type I and 972 Type II based on a BH-value significance threshold of 0.05.

The cancers HNSC, KICH, KIRC, and THCA yielded no significant Type I perturbagens, THCA yielded no significant Type II perturbagens, and KICH yielded no significant Type III perturbagens. The reasons for the absence of these perturbagen types from the respective cancers are not yet known and suggest new areas for future research to further define specific sex-associated differences in response to perturbagens in these cancer types. A complete listing of the number of Type I, II, and III drugs observed in each cancer can be found in Table 3, and a selection of top-ranking predictions is presented in Table 4. Results of the analysis in LIHC, LUAD and LUSC (which returned the greatest number of Type I and II perturbagens) are visualized as scatterplots in Fig. 3, and perturbagens showing strong Type I, Type II, and Type III patterns of connectivity with the input signatures are highlighted in each plot.

### Type I Perturbagen Predictions

According to our LincsCloud analyses, CDK1 knockdown is a predicted Type I perturbagen in LIHC, with a male connectivity score of 74.26 and a female connectivity score of −66.44. CDK1 inhibitors are being investigated as a potential treatment for cancers^10^, and our results suggest in LIHC that males may show the greatest benefit. The PTPN1 gene is part of the PTP family, involved in oncogenic transformation of cells in the mitotic cycle^11^, and PTP proteins are known to dephosphorylate epidermal growth factor^12^. Thus, PTPN1 knockdown would be expected to inhibit carcinogenesis and reduce tumor cell proliferation. According to our analyses, PTPN1 knockdown yielded a male connectivity score of 70.89 and a female score of −86.40; thus, we predicted that PTPN1 was also a Type I perturbagen, which would show the greatest benefit in male tumors.

### Type II Perturbagen Predictions

The SMAD3 gene is known to enhance invasion of HNSC via the transforming growth factor (TGF) beta^13^. In our analysis, SMAD3 knockdown is a Type II perturbagen, with a male connectivity score of −79.27 and a female score of 82.37, suggesting that it will show increased efficacy in females. In addition, we found the small-molecule compound midostaurin to be a predicted Type II perturbagen with a male score of −71.30 and a female score of 89.33.

### Type III Perturbagen Predictions

The LIHC Connectivity Map analysis predicted that etoposide and camptothecin are Type III perturbagens, each with connectivity scores of 98 or greater in both sexes. Both drugs^14^,^15^ have shown activity against human tumor cells. Both drugs are DNA topoisomerase inhibitors, suggesting that the efficacy of DNA topoisomerase inhibitors may not be dependent on sex.

## Discussion

Our study identified genes differentially expressed between tumors in male and females across multiple cancer types. We also identified pathways significantly associated with neoplastic progression in one or both sexes, some of which are potential treatment targets. Furthermore, we used a large-scale pharmacogenomics dataset (LincsCloud Connectivity Map) to develop a new, systematic approach to identify perturbagens that show significantly differential connectivity with male and female tumor expression signatures. Our study represents a large bioinformatics analysis of transcriptomic differences between male and female tumors and is the first study to use transcriptional differences between male and female tumors to predict novel male- and female-specific therapeutic strategies.

Several of our perturbagen predictions were supported by relevant literature. For example, our analyses predicted that tamoxifen in LUAD would be more efficacious in females as compared with males. This prediction agree with the findings of a 1997 study, in which the IC50 value of tamoxifen was found to be 0.32
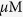
in a female cell line, compared to a range from 0.48
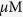
to 1.78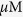 in male cell lines of the same ethnicity and similar age^16^. As estrogen receptors are more highly expressed in females than in males^17^, our prediction that tamoxifen, an estrogen receptor antagonist^18^, would be a Type II perturbagen is logical. Thus, our prediction method accurately and independently predicted sex-disparate sensitivity of a known Type II drug.

Our analysis predicted that everolimus in KIRC and a sorafenib-related drug (LSM-5610) in LIHC are Type III perturbagens. The everolimus prediction agrees with a 2010 study which found that sex was not a prognostic factor for survival during everolimus treatment in KIRC^19^. Our predictions also agree with the results of a study which found that the protein binding of sorafenib, thought to be responsible for its antitumor effects, was not correlated with sex^20^.

These examples represent only the tip-of-the-iceberg, and the vast majority of results and predictions generated from our analyses (linking sex to cancer molecular phenotype and drug response) have never before been evaluated and will require further external validation.

Our study has several limitations. One weakness is the relatively small amount of patient data within each cancer and sex for the tumor vs. normal analyses that informed the connectivity map analysis. Though data for each of the 7 cancers involved in drug predictions was collected from similar numbers of patients as a Phase II clinical trial, future work would ideally use transcriptomics data collected from tumor and normal samples from thousands of patients in each cancer, on the scale of Phase III clinical trials, thereby improving the accuracy of our predictions.

A second weakness lies in the lack of available gene expression data for normal samples for most patients in our analyses: out of the 17 cancers considered in our study, only 7 cancers had sufficient normal data for us to perform our sex-stratified tumor vs. normal differential gene expression analyses. Future work would ideally incorporate more normal gene expression data so that sex-differentiated gene expression patterns can be compared between tumors and normal samples in all 17 cancers.

A second weakness is the fact that our study is focused on using bioinformatics and statistical analyses of large-scale transcriptional and pharmacogenomic datasets to predict novel sex-specific therapeutic agents in cancer, but not on the validation of those predictions. The true value of the predictions generated from our work will be in:

1. Use of these data and analyses to drive future mechanistic studies in pre-clinical cancer models to dissect the influence of sex on molecular pathways and response to perturbagens.
2. Use of these data and analyses in the development of large-scale efforts to validate the most compelling and top-ranked predictions generated from our analyses in randomized clinical trials of cancer therapeutics.

We hope our study will lead to future studies to further define the role of sex in determining cancer molecular phenotypes and response to therapies, ultimately leading to the development of improved cancer diagnostics and therapeutics for all cancer patients.

## Materials and Methods

**Figure 1:**
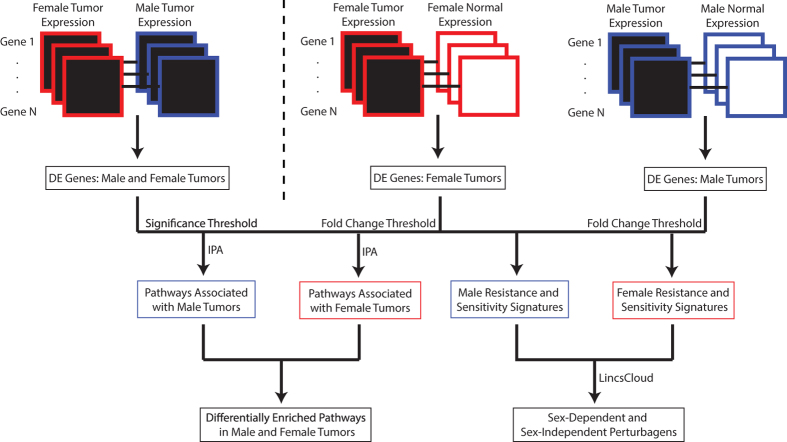
Study workflow. A flowchart depicting the inputs and outputs of the gene expression, pathway, and connectivity map analyses.

**Figure 2:**
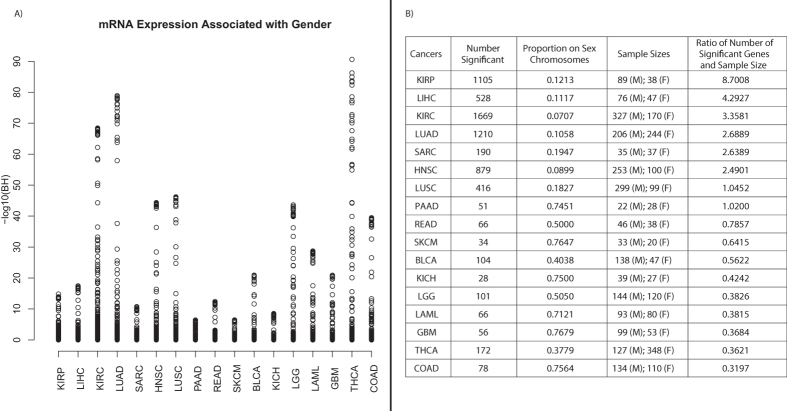
Tumor differential expression analysis with respect to sex. (**A**) Plot of all BH-values for genes in each of the 17 cancers. The cancers are listed in decreasing order of the ratio of number of identified significant genes to sample size on the x-axis. (**B**) Table of number of significant genes, sample size, and ratio of number of significant genes to sample size. The table includes entries for each of the 17 cancers.

**Figure 3:**
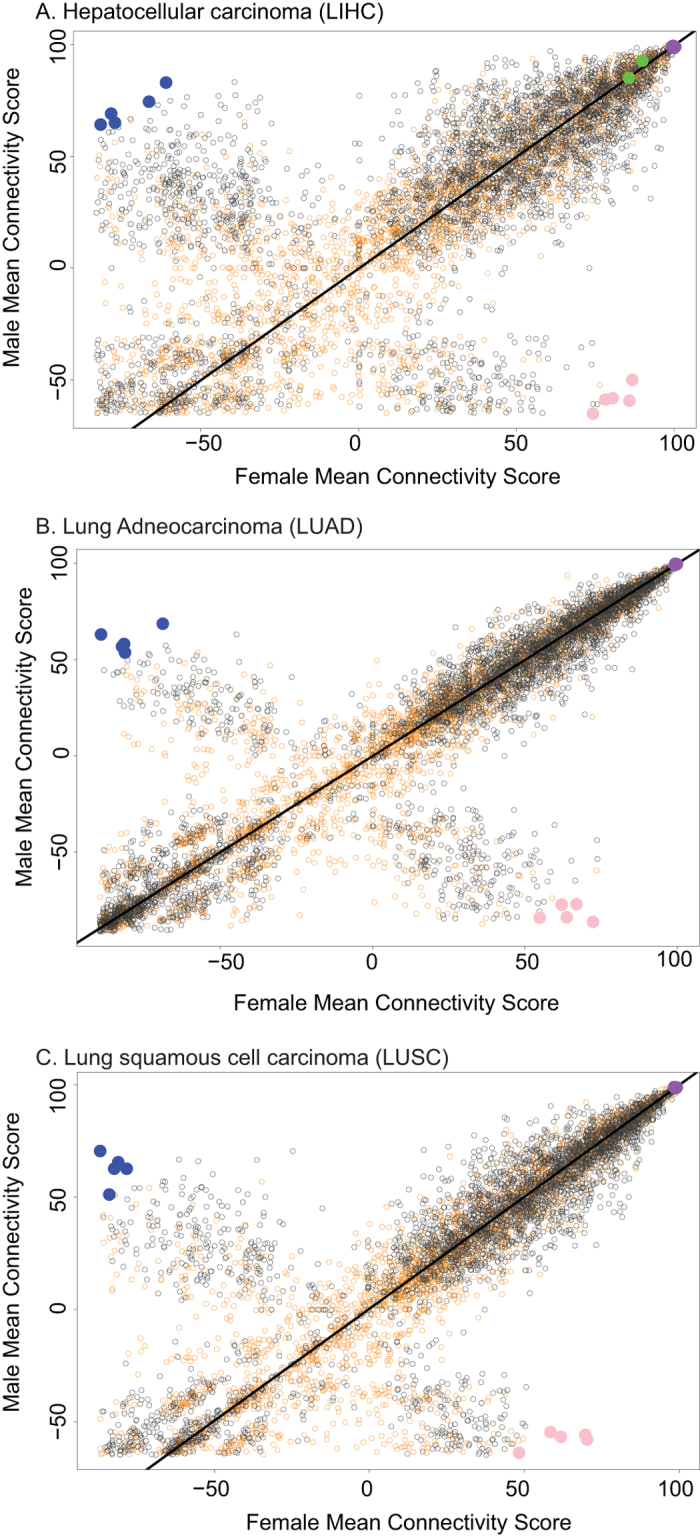
Male-Female Connectivity Score Scatterplots. Male-female distribution of connectivity scores of perturbagens (gene perturbations & drugs) in (**A**) LIHC, (**B**) LUAD, and (**C**) LUSC. These three cancers returned the largest number of sex-specific (Type I or II) perturbagens. Scores were obtained by querying the LincsCloud connectivity map for perturbagens which are positively associated with the sensitivity signature and negatively associated with the resistance signature for each cancer within each sex. Black points are genetic constructs (overexpression or knockdown); orange points are small-molecule compounds. Blue points are the five Type I perturbagens with greatest magnitude sex difference in connectivity score, pink points are the five Type II perturbagens with greatest magnitude sex difference in score, and violet points are the five Type III perturbagens with greatest sum of connectivity score. Green points indicate FDA-approved drugs.

An overview of the study work flow is given in Fig. 1.

### Cancer-Specific Transcriptomic Analyses With Respect to Sex

We obtained gene expression data from The Cancer Genome Atlas (TCGA) via the Broad Institute Genome Data Analysis Center (GDAC). We selected only the cancers with more than ten patients in each sex for analysis (Table S1). In total, gene expression data were collected from 2,352 male and 1,751 female patients. Gene expression levels were measured by TCGA using its RNAseq Version 2 algorithms. We used R scripts to coerce all data downloads into a format compatible with statistical tests and graphical visualizations.

We included genes of the sex chromosomes, as some of those genes unrelated to sex-specific functions may function in carcinogenesis^21^.

Using the R package “stats,” we applied the Wilcoxon Rank-Sum test to perform differential expression analyses. We used the Wilcoxon Rank-Sum test because it is nonparametric and thus more robust against outliers than the *t* test. We adjusted all *p*-values for multiple significance testing using the Benjamini-Hochberg method^22^, which yields a Benjamini-Hochberg (BH) value. To measure the magnitude of transcriptomic sex disparities, we also calculated cancer-specific fold changes of median gene expression levels for each gene.

### Cancer-Specific Transcriptomic Analyses with Respect to Tissue (Neoplastic vs. Normal)

We also aimed to analyze for sex-specific gene expression disparities between neoplastic and normal tissue. Thus, we applied the Wilcoxon Rank-Sum Test separately within each sex to compute cancer-specific *p-*values of gene expression disparities between neoplastic and normal tissue of the 7 cancer types with sufficient normal expression data (Fig. 1). We thereby identified genes with significantly differential expression between neoplastic and normal tissue within each sex.

Notably, TCGA only contained sufficient (defined as more than 10 cases in each sex) normal expression data in 7 of the 17 considered cancers. However, these 7 cancers thus have 40 cases total (10 male tumor, 10 female tumor, 10 male normal, 10 female normal); therefore, although each cohort is small, each corresponds to a patient cohort similar in size to that of a typical Phase II clinical trial^23^. Using this tumor-normal dataset, we applied the Wilcoxon rank sum test with respect to tissue type (tumor vs normal) within each sex, computing *p*-values of male and female tumor-normal gene expression disparities. We computed fold changes of such disparities. These were used to derive tumor and normal gene expression signatures within each sex, for use in Ingenuity Pathway Analysis and LincsCloud analyses.

### Pan-Cancer Transcriptomic Analyses

We aggregated cancer-specific *p*-values of transcriptomic sex disparities into pan-cancer *p*-values using Fisher’s method, applied through the R “MADAM” package. To quantify the magnitude of such disparities, we also computed fold changes of median gene expression across all cancers.

### Pathway Analyses

In this analysis, we considered the 7 cancers for which sufficient normal expression data was available in both sexes (Table S1). Separately within each sex, we considered genes with statistically significant (BH < 0.05) tumor-normal expression differences, as determined by our tumor-normal transcriptomics analyses. Using these significant genes and the corresponding tumor-normal BH values and fold changes, we used IPA to discover pathways significantly associated with the gene expression signature of tumor tissue (“tumor expression signature”) of each cancer within each sex. Within each cancer type, we then compared the pathways returned between the sexes to gauge sex differences in pathway association with the tumor expression signature.

### Connectivity Map Analysis

To construct male and female signatures for connectivity map analysis, we first separated the male and female samples. We identified genes differentially expressed in tumor vs. normal samples in males and in females. Of the genes significantly differentially expressed between tumor and normal samples, we selected the 250 genes with the largest fold change of expression difference to construct signatures for use in the connectivity map. We constructed resistance signatures using the 250 genes overexpressed in tumor tissue, and we constructed sensitivity signatures using 250 genes overexpressed in normal tissue. Similarly, we used pan-cancer tumor-normal fold changes to construct pan-cancer resistance and sensitivity signatures.

Separately within each sex, we obtained mean connectivity scores of thousands of perturbagens within each cancer by performing LincsCloud Connectivity Map analyses (http://www.lincscloud.org/) using cancer-specific resistance signatures as down-regulated signatures and cancer-specific sensitivity signatures as up-regulated signatures. Therefore, a strongly positive mean connectivity score indicated that the perturbagen significantly upregulated the sensitivity signature and downregulated the resistance signature, while strongly negative scores indicated the opposite. Thus a strongly positive mean connectivity score for a perturbagen in one sex indicated that tumor cells of that sex were predicted to be sensitive to the perturbagen; conversely, a strongly negative mean connectivity score indicated that tumor cells of that sex were predicted to be resistant to the perturbagen.

### Permutation Analysis

The goal of these permutation analyses was to estimate the statistical significance of male-female differences in connectivity scores generated by our sex-stratified Connectivity Map analyses. This approach was modeled on the PMASE method developed by Liu *et al.*^9^.

Using the raw TCGA data in the seven cancers for which we performed LincsCloud analyses, we conducted *N* = 1,000 permutations of the sex labels for each of the patients within each cancer. Thus, each patient was effectively randomly assigned a sex, while the total number of patients of each sex were held constant. Each permutation was therefore equivalent to a sample under the null hypothesis that there was indeed no genetic difference between the sexes and thus no underlying basis for observed sex differences in connectivity score.

For each permutation, we computed the resistance and sensitivity signatures using the methods described earlier. We then queried LincsCloud for perturbagens which could upregulate our sensitivity signature and downregulate our resistance signature, using UNIX shell scripts and a command line interface provided by LincsCloud known as LincsCloud Compute Connectivity on the Cloud (C3). These queries yielded sex-specific connectivity scores for each perturbagen for *N* = 1000 permutations within each of the 7 cancers considered in drug predictions as well as our pan-cancer analysis. Within each cancer-specific as well as our pan-cancer analyses, we then computed the number of perturbagens, out of the total of *N* = 1000, which yielded a sex difference in connectivity score greater in magnitude than the difference we obtained through our actual analyses. This fraction represents the empirical probability of obtaining a connectivity score difference greater than the one observed under the null hypothesis and is thus the raw empirical p-value for the observed sex difference in connectivity score. We corrected these raw *p*-values for multiple hypothesis testing using the method of Benjamini and Hocbherg.

*Code Availability*: Code for reproducing analyses can be accessed from the website accompanying the manuscript http://becklab.github.io/SexLinked.

## Additional Information

**How to cite this article**: Ma, J. *et al.* Systematic Analysis of Sex-Linked Molecular Alterations and Therapies in Cancer. *Sci. Rep.* **6**, 19119; doi: 10.1038/srep19119 (2016).

## Supplementary Material

Supplementary Table S1

## Figures and Tables

**Table 1:** Summary of Pathway Analysis Results.

Cancer	Number of Pathways Enriched in Males Only	Number of Pathways Enriched in Females Only	Number of Pathways Enriched in Both	Proportion of Significant Pathways Enriched in Only One Sex
LUAD	115	36	28	0.84
KICH	96	27	37	0.77
LIHC	170	21	59	0.76
HNSC	97	57	101	0.6
KIRC	75	78	159	0.49
Pan-Cancer	69	22	123	0.43
THCA	45	51	148	0.39
LUSC	29	22	185	0.22

This table details the number of pathways found enriched (P < 0.05) in male, female, or both tumors in each of the 7 cancers considered. The proportion discordant measures the proportion of pathways enriched significantly in only one sex out of all pathways significantly enriched.

**Table 2:** Sex-disparate pathway enrichment. Selection of pathways significantly enriched in tumors of one sex but not the other.

Pathway	Female p-value	Male p-value	Cancer
EIF2 Signaling	Non-significant	2.63E-06	HNSC
Tight Junction Signaling	Non-significant	1.07E-05	HNSC
Mitochondrial Dysfunction	Non-significant	1.26E-05	HNSC
LPS/IL-1 Mediated Inhibition of RXR Function	Non-significant	1.58E-05	HNSC
Activation of IRF by Cytosolic Pattern Recognition Receptors	0.00112	Non-significant	HNSC
Crosstalk between Dendritic Cells and Natural Killer Cells	0.0239	Non-significant	HNSC
Coagulation System	Non-significant	0.000257	KICH
Acute Phase Response Signaling	Non-significant	0.00049	KICH
Fatty Acid Î^2^-oxidation I	Non-significant	1.78E-05	KIRC
Ketogenesis	Non-significant	1.48E-04	KIRC
Valine Degradation I	Non-significant	3.3E-04	KIRC
LXR/RXR Activation	Non-significant	5.01E-13	LIHC
FXR/RXR Activation	Non-significant	2.00E-11	LIHC
LPS/IL-1 Mediated Inhibition of RXR Function	Non-significant	7.94E-08	LIHC
Coagulation System	Non-significant	4.90E-07	LIHC
Valine Degradation I	Non-significant	1.15E-06	LIHC
Sonic Hedgehog Signaling	0.0468	Non-significant	LIHC
Axonal Guidance Signaling	Non-significant	9.33E-05	LUAD
Role of Macrophages, Fibroblasts and Endothelial Cells in Rheumatoid Arthritis	Non-significant	1.02E-04	LUAD
IL-8 Signaling	Non-significant	1.62E-04	LUAD
EIF2 Signaling	9.12E-05	Non-significant	LUAD

**Table 3:** Number of significant Type I, II, and III perturbagens found in each cancer.

Cancer	Type I	Type II	Type III	Total Sex-Specific
LIHC	432	207	13	639
LUAD	175	243	134	418
LUSC	214	153	153	367
KIRC	0	312	1	312
HNSC	0	236	33	236
KICH	0	232	0	232
THCA	0	0	3	0

The table summarizes the number of significant perturbagens of each type in each cancer, based on an empirical BH-value threshold of 0.05 and an additional significance threshold magnitude of 90 for Type III drugs only. BH-values were calculated through permutation analysis (see Materials and methods). The “total sex-specific” column indicates the number of sex-specific (that is, Type I and II) perturbagens in each cancer.

**Table 4:** Connectivity Scores and Permutation BH-values of Novel Predicted Type I, II, and III Perturbagens.

Perturbagen	Cancer	Male Score	Female Score	Predicted Type	BH-value
CDK1 knockdown	LIHC	74.26	−66.44	I	<0.0001
PTPN1 knockdown	LUSC	70.89	−86.40	I	<0.0001
SMAD3 knockdown	HNSC	−79.27	82.37	II	<0.0001
HSPA4 knockdown	HNSC	−80.84	78.58	II	<0.0001
Midostaurin	All	−71.30	89.33	II	<0.0001
Etoposide	LIHC	98.76	99.70	III	0.9575
Camptothecin	LIHC	98.97	99.25	III	0.9498

The table summarizes several predictions for sex-dependent and sex-independent perturbagens. The BH-value quantifies the statistical significance of the male-female connectivity score difference, as computed by our permutation analyses (see Materials and Methods). “All” indicates that the perturbagen prediction was made on pan-cancer resistance and sensitivity signatures.
