# Supplementary material for: Systematic Analysis of Sex-Linked Molecular Alterations and Therapies in Cancer
Source: Sci Rep. 2016 Jan 12;6:19119. doi: 10.1038/srep19119 (PMC4709570; doi:10.1038/srep19119)
Supplement: Supplementary Table S1 [file srep19119-s1.pdf]

Title: Systematic Analysis of Sex-Linked Molecular Alterations and Therapies in Cancer

Authors: Jonathan Ma, Sadhika Malladi, Andrew H Beck

Supplementary Materials

BLCA	Bladder Urothelial Carcinoma	COAD	Colon Adenocarcinoma	GBM	Glioblastoma Multiforme
HNSC	Head & Neck Squamous Cell Carcinoma	KICH	Kidney Chromophobe	KIRC	Kidney Renal Clear Cell Carcinoma
KIRP	Kidney Renal Papillary Cell Carcinoma	LAML	Acute Myeloid Leukemia	LGG	Brain Lower Grade Glioma
LIHC	Liver Hepatocellular Carcinoma	LUAD	Lung Adenocarcinoma	LUSC	Lung Squamous Cell Carcinoma
PAAD	Pancreatic Adenocarcinoma	READ	Rectal Adenocarcinoma	SARC	Sarcoma
SKCM	Skin Cutaneous Melanoma	THCA	Thyroid Carcinoma		

Table S1. Cancers Analyzed in Cancer-Specific Transcriptomic Analyses and Corresponding Abbreviations. Each of these cancers included neoplastic gene expression data for at least 10 patients of each sex and thus was included in our transcriptomic analyses. The cancers highlighted in yellow included additional nonneoplastic gene expression data for at least 10 patients of each sex and thus were included in our pathway and connectivity map analyses.
